# The safety of SGLT-2 inhibitors in diabetic patients submitted to elective percutaneous coronary intervention regarding kidney function: SAFE-PCI pilot study

**DOI:** 10.1186/s13098-023-01107-9

**Published:** 2023-06-26

**Authors:** Mateus Paiva Marques Feitosa, Eduardo Gomes Lima, Alexandre Antônio Cunha Abizaid, Roxana Mehran, Neuza Helena Moreira Lopes, Thiago de Assis Fischer Ramos, Alexandre Hideo-Kajita, Roberto Kalil Filho, Carlos Vicente Serrano Junior

**Affiliations:** 1 Instituto Do Coração (InCor), Hospital das Clínicas HCFMUSP, Faculdade de Medicina, Universidade de São Paulo, São Paulo, Brazil; 2 Icahn School of Medicine at Mount Sinai, New York, USA; 3 Av. Dr. Eneas de Carvalho Aguiar 44, Departamento de Aterosclerose, 2nd Floor, Cerqueira César, São Paulo, SP 05403–000 Brazil

**Keywords:** SGLT2 inhibitors, Coronary artery disease, Percutaneous coronary intervention, Acute kidney injury, Contrast-induced nephropathy

## Abstract

**Background:**

Percutaneous coronary intervention (PCI) is one of the most performed well-succeeded therapeutic procedures worldwide, reducing symptoms and improving quality of life. Neutrophil Gelatinase-associated Lipocalin (NGAL) is a biomarker of acute kidney injury (AKI) produced early after an ischemic renal insult. Osmotic diuresis and the vasoconstriction of the afferent arteriole promoted by Sodium-glucose Cotransporter-2 Inhibitors (SGLT2i) generate a concern regarding the possibility of dehydration and consequent AKI. There is no consensus on the maintenance or discontinuation of SGTL2i in patients who will undergo PCI. This study aimed to evaluate the safety of empagliflozin in diabetic patients submitted to elective PCI regarding kidney function.

**Methods:**

SAFE-PCI trial is a prospective, open-label, randomized (1:1), single-center pilot study and a follow-up of 30 days. The SGLT2i empagliflozin 25 mg daily was initiated at least 15 days before PCI in the intervention group and maintained until the end of the follow-up period. Serum NGAL was collected 6 h after PCI and creatinine before PCI, 24 h, and 48 h after the procedure. As per protocol, both groups received optimal medical treatment and standard protocol of nephroprotection.

**Results:**

A total of 42 patients were randomized (22 patients in the iSGLT-2 group and 20 patients in the control group). There was no difference between-group baseline data. The primary outcome (NGAL and creatinine values post PCI) did not differ in both groups: the mean NGAL value was 199 ng/dL in the empagliflozin group and 150 ng/dL in the control group (*p* = 0.249). Although there was an initial increase in creatinine in the SGLT-2i group compared to the control group between baseline creatinine and pre-PCI and 24 h post-PCI creatinine, no difference was detected in creatinine 48 h post-PCI (*p* = 0.065). The incidence of CI-AKI, determined by KDIGO criteria, in the iSGLT2-group was 13.6% and 10.0% in the control group without statistical difference.

**Conclusion:**

The present study showed that the use of empagliflozin is safe regarding kidney function during elective PCI in patients with T2D when compared with no use of SGLT2i.

*Trial registration* Our clinical study is registered on ClinicalTrials.gov with the following number: NCT05037695.

## Keypoints

- Question: Should we maintain or discontinue SGTL2 inhibitors in diabetic patients who will undergo percutaneous coronary intervention?
- Findings: The present study showed that the use of SGLT2i (empagliflozin) is safe regarding kidney function during elective PCI in patients with T2D when compared with no use of SGLT2i.
- Meaning: With published data on renal safety of empagliflozin pre-PCI in a pilot study, clinical outcomes such as CI-AKI can now be evaluated in a larger randomized clinical trial based on an adequate sample size calculation.

## Background

There is established benefit between PCI and stable coronary artery disease (CAD) patients, by reducing symptoms and improving quality of life [1]. Still, type 2 diabetes (T2D) is present in 25% of patients undergoing elective PCI and represents the main risk factor for the onset of acute kidney injury (AKI) after PCI [1, 2]—mainly due to the use of contrast medium [1, 2]. Contrast-induced AKI (CI-AKI) is considered as a new-onset or an exacerbation of chronic renal dysfunction following the administration of contrast media dye, without other potential causes [2].

Neutrophil Gelatinase-associated Lipocalin (NGAL) is a biomarker of AKI produced early after an ischemic renal insult. The practice of determining the biomarker levels has been validated in previous publications, either in experimental or clinical studies [3, 4]. This includes the detection of the development of CI-AKI at least 24 h before the increase in serum creatinine. CI-AKI is related to increased death, progression of kidney disease, need for dialysis, and increased health-related costs [2, 5]. Although, a myriad of strategies were previously tested aiming to reduce CI AKI with inconsistent results, such as statins, n-acetylcysteine, and sodium bicarbonate. Up to now, the only effective therapy for CI AKI prevention, is intravenous hydration, with isotonic saline solution and the use of low- or iso-osmolar contrast media [5].

Sodium-glucose cotransporter 2 inhibitors (SGLT2i) reduced the rate of hyperglycemia in patients with T2D by decreasing renal glucose reabsorption, thus increasing urinary glucose excretion [6]. SGLT2i have demonstrated a protective clinical effect in T2D patients with high cardiovascular risk, by reducing mortality rate, hospitalization due to heart failure, and a decrease in the progression of kidney disease [6–8]. On the other hand, the osmotic diuresis and the vasoconstriction of the afferent arteriole promoted by SGLT2i generate a concern regarding the possibility of dehydration and consequent AKI [9].

Real-world evidence studies suggest that patients using SGLT2i, including empagliflozin, developed less AKI and displayed less overall renal decline when compared to other glucose-lowering agents [6]. This discloses the likely renoprotective properties of SGLT2i counter to AKI for patients with T2D. The role of SGLT2i in reducing the risk of CI-AKI in T2D undergoing elective PCI is to be seen.

In SAFE-PCI trial, we evaluated if SGLT2i (empagliflozin) prevents CI-AKI among T2D patients undergoing elective PCI, as expressed by blood levels of biomarkers NGAL and creatinine. Our study intented to explore the specific effects of empagliflozin on the extent of CI-AKI among T2D patients with stable CAD undergoing PCI.

## Methods

### Trial design

SAFE-PCI trial is a pilot, single-center, prospective, open-label, interventional controlled randomized (1:1) study aiming to assess AKI up to 48 h after PCI. NGAL and creatinine changes were measured after using oral empagliflozin 25 m/day for at least 15 days before the procedure. The SGLTi group was compared to a control group, of stable CAD and T2D patients on optimal medical therapy and without SGLT2i.

The exclusion criteria were: estimated glomerular filtration rate (eGFR) < 30 mL/min/1.73m^2^ or dialysis therapy; acute coronary syndrome in the last 30 days; need for urgent or emergency PCI; use of non-steroidal anti-inflammatory drugs in the last 30 days before randomization; known pregnancy; and inability to sign the consent form.

**Fig. 1.**
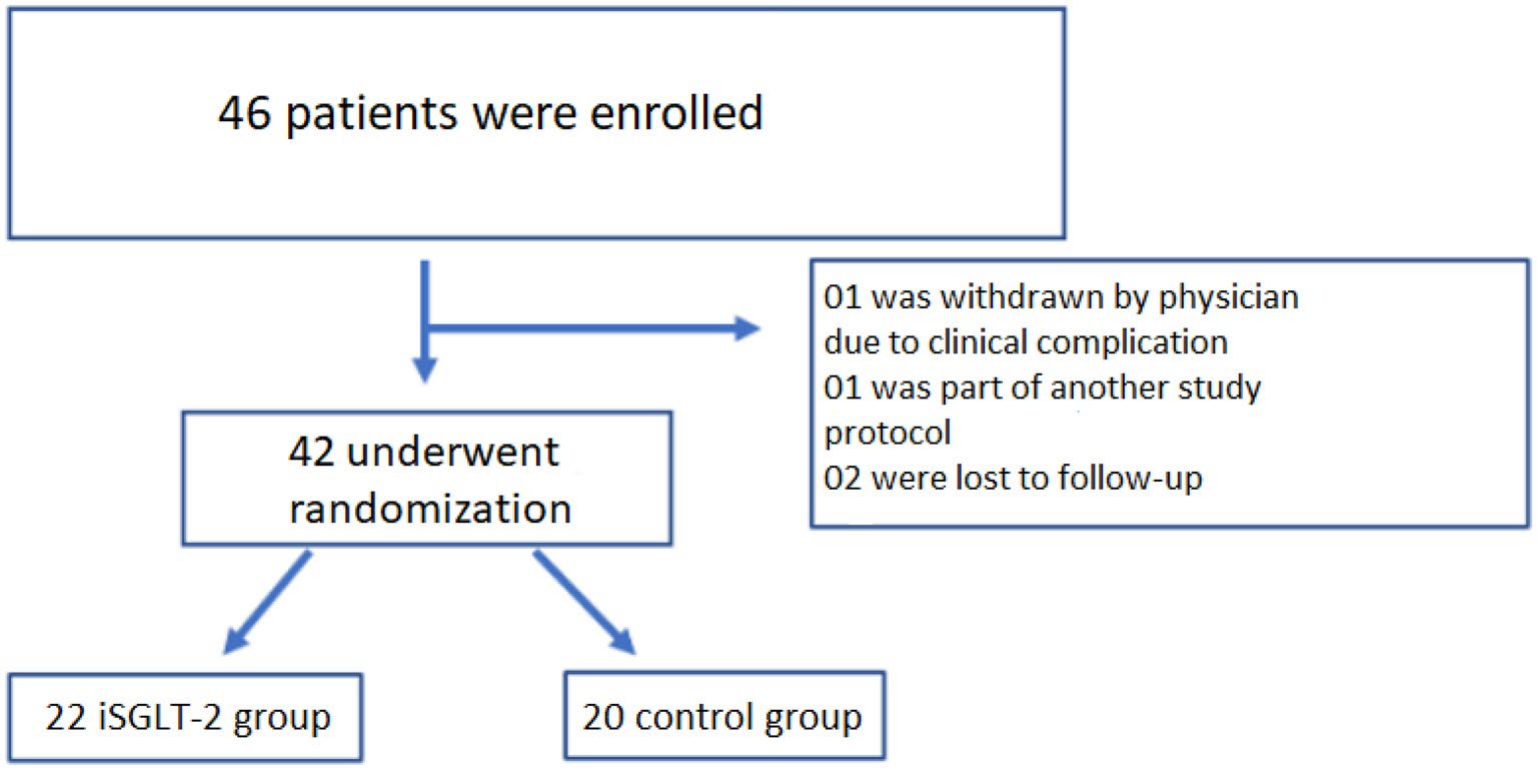
Study flow showing patient enrollment and randomization

The patients were all admitted to the general cardiology ward of our institution and underwent coronary angiography (CAG) and PCI from August, 2021 to June, 2022. The study flow diagram presenting the enrolment and randomization data is seen in Fig. 1. For this study, those patients with a diagnosis of T2D and CAD and available serum creatinine measurements during hospitalization were included. As a pilot study, there were no preliminary data of CI-AKI incidence in patients submitted to PCI, and the sample size was estimated in 46 patients evaluated for inclusion. After excluding 4 patients, 42 patients were enrolled and randomized to the SAFE-PCI study, either the SGLT2i group (n = 22) or to the control group (n = 20). All 42 patients were followed until the end of the study.

This study was approved by the institutional review board of the Instituto do Coracao (InCor) of the Faculty of Medicine of the University of Sao Paulo and patients needed to sign the consent form before recruitment.

### Procedural aspects

PCI consisted of the use of drug-eluting stents only, due to its already established benefits for the T2D population [10].

The standard dose of acetylsalicylic acid 100 mg/day was maintained. All patients received a second antiplatelet drug on pre-PCI at the hospital admission in a loading dose for those who were already not on dual antiplatelet therapy, maintaining the following maintenance dosis either: clopidogrel 75 mg/day, ticagrelor 90 mg/twice a day or prasugrel 10 mg/day, according to institutional availability.

In all cases, renoprotection strategies were used to prevent CI-AKI, counting low-osmolar or iso-osmolar contrast media dye and hydration with intravenous isotonic crystalloid solutions (1 ml/kg/hour 6 h before PCI), as well as the restraint of nephrotoxic agents. Intravascular imaging was used, when available [11].

### Blood sample collection

**Fig. 2.**
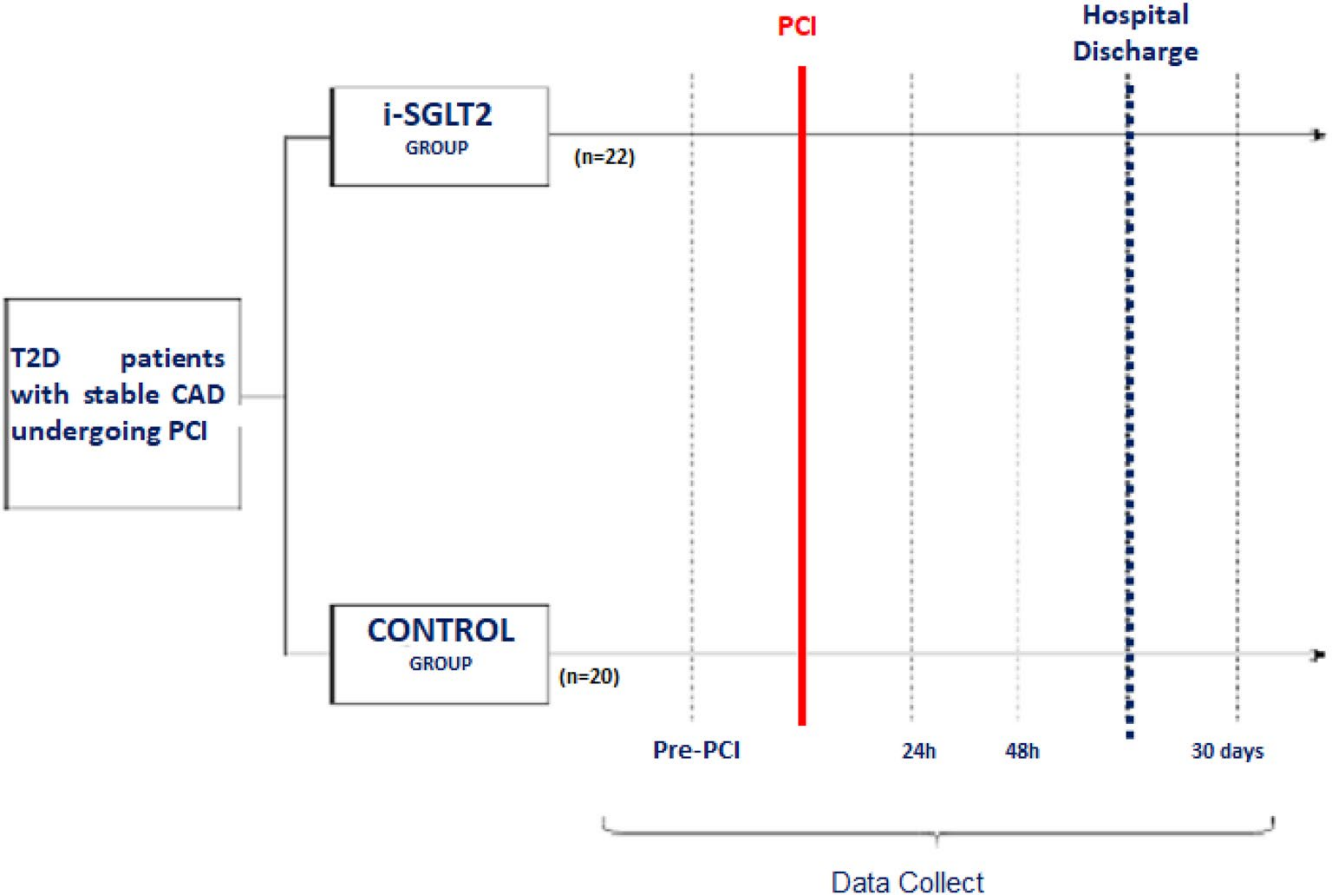
Study protocol

Serum NGAL was collected 6 h after the procedure. Serum creatinine was collected at the time of randomization, before and after PCI at pre-specified intervals of 6, 24, and 48 h, and at 30 days (see Fig. 2). Laboratory tests, including hemograms, myocardial necrosis markers, lipid profiles, kidney function tests and glycemic blood samples were collected before and after PCI.

### Definitions

T2D was defined according to American Diabetes Association (ADA) guidelines [12]. Essentially, T2D was confirmed by fasting glucose levels (≥ 126 mg/dL) or by a previous diagnosis when the patient was on oral hypoglycemic agents or insulin [12].

CAD was defined as a narrowing of at least 50% luminal diameter in at least one major coronary artery, evaluated by two independent experienced interventional cardiologists.

A SYNTAX score value is the sum of the points assigned to each individual lesion identified in the coronary tree with > 50% diameter narrowing in vessels > 1.5 mm diameter evaluated by a coronary angiogram to determine the complexity of coronary artery disease. The higher the score, the greater the coronary artery disease complexity. All coronary angiographies were evaluated by an experienced interventional cardiologist and SYNTAX score was calculated and described in angiographic features [10].

CI-AKI is the third most frequent cause of acute kidney dysfunction and is defined as a 25.0% increase in baseline creatinine or an absolute increase of 0.5 mg/dL between 48 and 72 h after contrast use according to the Kidney Disease Improving Global Outcomes (KDIGO) classification [13].The individual risk of developing CI-AKI can be estimated using the Mehran Score, which consists of risk stratification. An increased Mehran Score confers an exponentially increased risk of CI-AKI, ranging from 8.4 to 55.9% [14].

Complications such as spontaneous myocardial infarction (MI) was defined by the 4th universal definition of myocardial infarction [15] and periprocedural MI (PMI) according to Society for Cardiovascular Angiography and Interventions (SCAI) definition [16]. Bleeding was defined by Bleeding Academy Research Consortium (BARC) definition [17].

### Outcomes

The primary outcome of the study was serum NGAL measurements 6 h after the procedure.

Secondary outcomes considered the individual occurrences of: creatinine level changes (at the pre-specified time-points); CI-AKI; PMI; spontaneous MI; stroke; definite or probable stent thrombosis; major bleeding, namely BARC from 3 to 5; and all-cause mortality.

### Statistical analysis

Quantitative variables were presented as average and standard deviation (SD), or median and interquartile ranges (IQR). Qualitative variables were expressed as absolute values and frequency of occurrences. The distribution of continuous variables was performed by the Kolmogorov–Smirnov test.

The comparison of two means for the quantitative variables was performed using Student's t-test. When normality was rejected (non-parametric distribution), the Mann–Whitney U test was used. The Chi-square test was used to compare qualitative variables in groups. The Fisher exact test was used for categorical variables.

One-way ANOVA with repeated measures was used to compare creatinine levels in different times in each group.

Logistic regression analysis was used to establish the risk of occurrence of events between groups.

The tests were two-tailed and performed with a significance level of 5%. SPSS version 21.0 software for Macintosh (IBM^®^ SPSS Statistics, Armonk, NY, USA) was used for statistical analysis. Randomization was performed using the REDCap—HCFMUSP program v11.0.3 (Vanderbilt University, Nashville, TN, USA).

## Results

### Baseline characteristics and medication use

Except for HDL and CKMB levels with a *p*-value of 0.037 and 0.016, respectively, even though their values remained in the range of normality (see Table 1), there were no differences in baseline clinical and laboratory characteristics between the groups. No differences were observed between the groups regarding insulin use, hypoglycemic and anti-ischaemic medications (see Table 2).

**Table 1:** Baseline clinical and laboratory characteristics of the groups

	SGLT2i (n = 22)	Control (n = 20)	*p*-value
Age (years)	65 ± 10	64 ± 6	0.82
Male sex	14 (63%)	15 (75%)	0.42
Arterial hypertension	19 (86%)	14 (70%)	0.83
Active smoker	12 (54%)	9 (45%)	0.41
Previous stroke	5 (22%)	2 (10%)	0.26
Peripheral arterial disease	2 (9%)	1 (5%)	0.60
eGFR (mL/min/1.73m^2^)	62.10 ± 22.50	68.20 ± 17.70	0.10
Mehran score	6.70 ± 3.68	5.55 ± 2.09	0.44
LVEF (%)	50 ± 14%	60.00 ± 9.16	0.18
Prior PCI (%)	5 (22%)	3 (15%)	0.52
Prior CABG (%)	4 (18%)	2 (10%)	0.44
Hemoglobin (g/dL)	11.8 ± 4.6	12.5 ± 4.0	0.50
Platelets (mm^3^)	220,000 ± 71,554	209,750 ± 41,079	0.23
Troponin (ng/mL)	56 ± 101	24 ± 43	0.18
CKMB (ng/mL)	2.32 ± 1.37	1.32 ± 0.75	0.016
Glycated hemoglobin (%)	6.3 ± 3.0	7.6 ± 2.0	0.16
Urea (mg/mL)	47.68 ± 17.00	45.10 ± 31.20	0.64
Creatinine (mg/mL)	1.24 ± 0.35	1.06 ± 0.26	0.24
Total cholesterol (mg/mL)	178 ± 55	160 ± 46	0.16
HDL-cholesterol (mg/mL)	47 ± 13	40 ± 9	0.037
LDL-cholesterol (mg/mL)	98 ± 41	96 ± 40	0.92
Triglycerides (mg/mL)	148 ± 63	144 ± 55	0.87

eGFR, estimated glomerular filtration rate; LVEF, left ventricular ejection fraction; PCI, percutaneous coronary intervention; and, CABG, coronary artery bypass graft

T student was used to compare means between groups

Chi square test was used to compare proportion between groups

**Table 2:** Baseline medications

	SGLT2i (n = 22)	Control (n = 20)	p-value
Insulin	9 (40.9%)	5 (25%)	0.27
Metformin	20 (90.9%)	18 (90%)	0.92
Sulfonylurea	5 (22.7%)	9 (45%)	0.12
DPP-4 inhibitor	0 (0%)	1 (5%)	0.28
Glitazone	0 (0%)	0 (0%)	–
Oral anticoagulant	4 (18.2%)	1 (5%)	0.18
Statin	21 (95.5%)	20 (100%)	0.33
Beta-blocker	19 (86.4%)	16 (80%)	0.58
Angiotensin II receptor blocker	4 (18.2%)	9 (45%)	0.06
ACE inhibitors	15 (68.2%)	9 (45%)	0.12

DDP, dipeptidyl peptidase 4; ACE, angiotensin-converting enzyme. Chi square test was used to compare proportion between groups

### Angiographic characteristics

Baseline angiographic characteristics are shown in Table 3. There was no difference between groups regarding the SYNTAX score (*p* = 0.11), the number of implanted stents (*p* = 0.20), stent diameter (*p* = 0.55), and contrast dye volume (*p* = 0.23).

**Table 3:** Angiographic features

	SGLT2i (n = 22)	Control (n = 20)	p-value
Location of lesions			
LMCA	0	1	0.28
LAD	12	14	0.08
LCx	11	4	0.043
RCA	5	7	0.10
VG	0	1	0.28
Bifurcation lesion	14	7	0.054
Stent diameter (mm)	40 ± 23	61 ± 30	0.551
Number of stents placed	28	27	0.20
SYNTAX score	16.60 ± 7	14.05 ± 9	0.11
Contrast medium volume (mL)	144 ± 66	176 ± 54	0.23
Procedural time (min)	60 ± 30	77 ± 40	0.46
ACC/AHA Lesion classification	A-5	A-2	0.08
	B1–6	B1–6	
	B2–5	B2–9	
	C-6	C-4	

ACC, American College of Cardiology; AHA, American Heart Association; LMCA, left main coronary artery; LAD, left anterior descending artery; LCx, left circumflex artery; RCA, right coronary artery; VG, vein graft. T student was used to compare means between groups

One patient in the SGLT2i group underwent balloon angioplasty only to due unsuccessful stent implantation.

### Outcomes

**Fig. 3:**
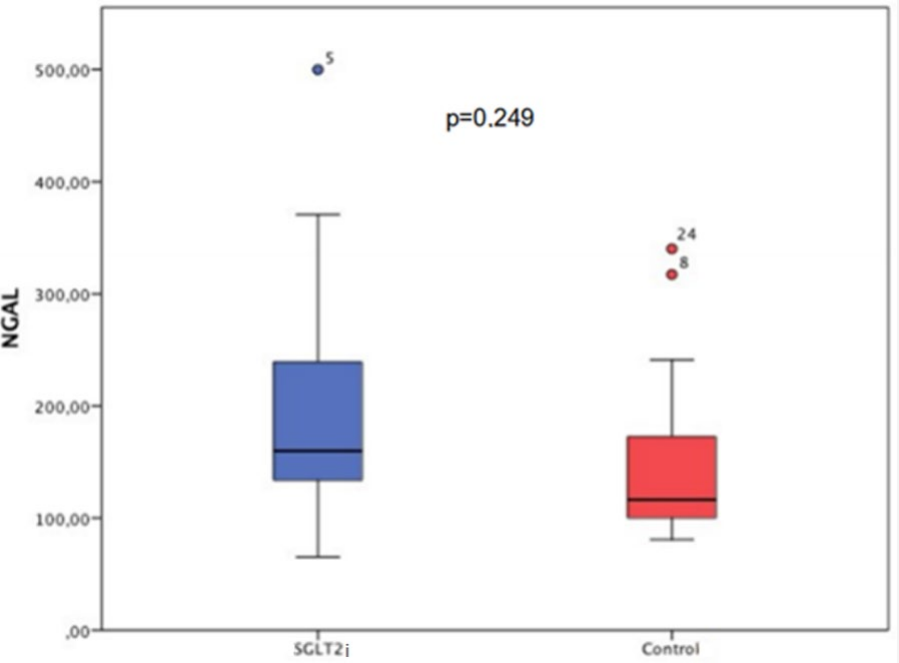
NGAL value (ng/dL) 6 h after percutaneous coronary intervention. There was no difference in the primary endpoint of the study. Mean serum NGAL 6 h after PCI was 199 ng/dL in the SGLT2i group and 150 ng/dL in the control group (*p* = 0.249). *Test-t used to compare two different groups

There was no difference in the primary endpoint of the study (see Fig. 3). Mean serum NGAL 6 h after PCI was 199 ng/dL in the SGLT2i group, and 150 ng/dL in the control group (*p* = 0.249).

**Fig. 4:**
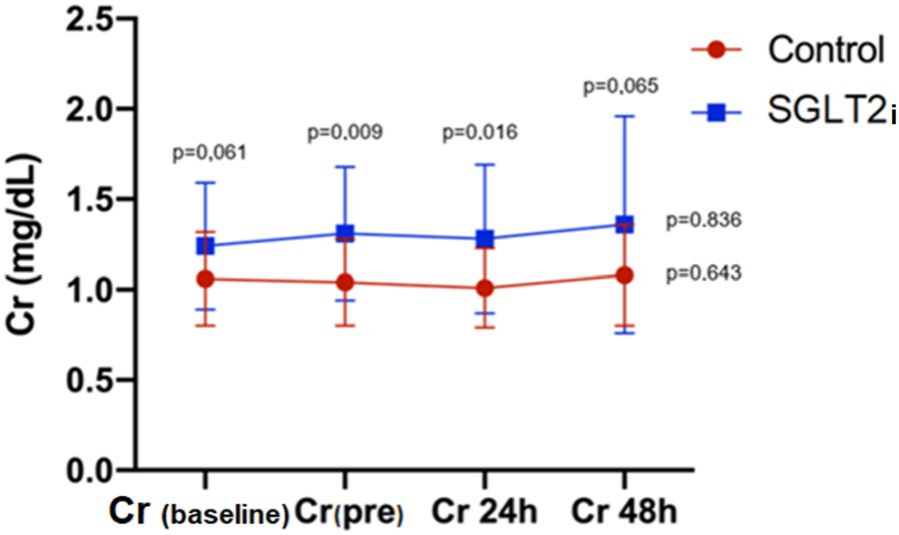
Creatinine levels at pre-specified time-points. There was no difference in baseline creatinine levels (*p* = 0.061) between groups. In the SGLT2i group, an absolute increase in creatinine from baseline to creatinine levels at pre-PCI and 24 h after PCI was observed when compared to the Control group. However, the difference between groups after 48 h of PCI was not statistically significant. *Test-t used to compare two creatinine levels in different groups. **One-way ANOVA with repeated measures was used to compare creatinine levels in different times in each group

As seen in Fig. 4, there was no difference in baseline creatinine levels (*p* = 0.061) between groups. In the SGLT2i group, an absolute increase in creatinine from baseline to creatinine levels at pre-PCI and 24 h after PCI was observed when compared to the Control group (*p* = 0.009 and 0.016, respectively). However, the difference between groups after 48 h of PCI is no longer statistically significant (*p* = 0.065).

**Fig. 5:**
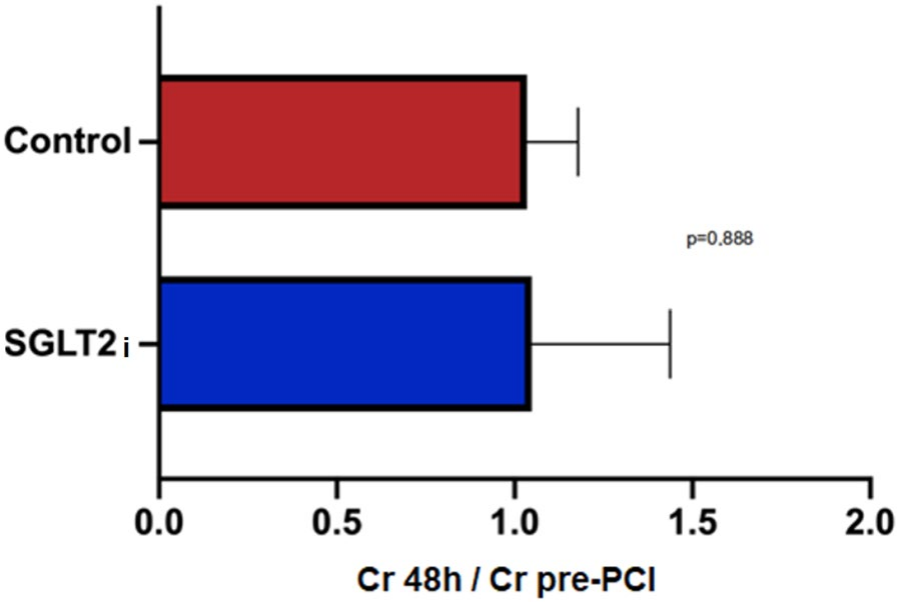
There was no difference between the groups in the ratio of creatinine at 48 h and creatinine before PCI. PCI, percutaneous coronary intervention. *Test-t used to compare two creatinine levels in different groups

The ratio of creatinine at 48 h and creatinine before PCI did not show a difference between groups (*p* = 0.888) (see Fig. 5).

The incidence of CI-AKI was similar in both groups, occurring in 3 patients in the SGLT2i group (13.6%) and 2 patients in the control group (10%) (*p* = 0.71). The the same result was observed regarding the occurrence of PMI with 26% in the SGLT2i group vs. 30% in the Control group (*p* = 0.59).

A multivariate sensitivity analysis of the predictors of CI-AKI was performed. The Mehran score was found as the only independent variable to predict the occurrence of CI-AKI (Harzard Ratio: 1.37; 1.02–1.85; *p* = 0.033). The isolated serum value of NGAL 6 h after PCI did not correlate with the occurrence of CI-AKI.

One patient in the SGLT2i group had a hemorrhagic stroke as well as an AKI that required hemodialysis. In the 30-day analysis, there were no episodes of major bleeding, stent thrombosis or death. Other patient in the SGLT2i group presented a coronary perforation without hemodynamic instability during and after the procedure.

## Discussion

In our trial, the use of empagliflozin before PCI did not increase the occurrence of AKI in patients with T2D and stable CAD undergoing PCI through serum NGAL value and creatinine curve. In addition, there was similar rates of CI-AKI between groups.

These observations are consistent with previously reported data from non-randomized clinical trials. Despite the kidney injury after the use of contrast media, CI-AKI rates did not raise after PCI when associated with the use of SGLT2i. In addition, to the best of our knowledge, SAFE-PCI was the first trial to evaluate kidney injury in the context of PCI through assessment of NGAL, as an early predictor of CI-AKI, which is a biomarker more accurate than serum creatinine.

Part of the benefits of SGLT2i is attributed to the reduction in blood pressure, weight, and albuminuria [18–20]. A recently published meta-analysis, including data from the three largest randomized studies with this therapeutic class (DECLARE, EMPA REG, and CANVAS), pointed to a 44% reduction in the incidence of AKI in the population using SGLT2i compared to the control group [21]. Although, SAFE-PCI is a pilot study, it is the first randomized trial evaluating the impact of SGLT2i in peri-procedural status of stable CAD and T2D patients undergoing to PCI.

The reduction of Na+ reabsorption by the kidney is associated with SGLT2 inhibition with consequent reduction of Na+/K+ ATPase activity in the proximal tubule seems to be related to greater perfusion and oxygenation in the renal cortex, as well as a greater tolerance to ischemia and reperfusion injury [22]. Part of a potential benefit in preventing AKI could be explained by these mechanisms. Moreover, an anti-inflammatory mechanism of dapagliflozin is also postulated from the reduction of nuclear factor kappa-light-chain-enhancer of activated B cells (NF-Kβ), reducing AKI [21].

The tubuloglomerular feedback system is an adaptive mechanism through which the reabsorption of sodium and chloride in the macula densa promotes the release of adenosine, contributing to the vasoconstriction of the afferent arteriole [20]. In diabetes, as a result of increased reabsorption of sodium and chloride in the proximal tubule, delivery to the macula densa is decreased, leading to less reabsorption of solutes and a consequent decrease in adenosine production. By promoting relative afferent arteriolar vasodilation, this mechanism contributes to glomerular hyperperfusion, hypertension and hyperfiltration in diabetes [20, 23, 24].

By blocking the reabsorption of NaCl in the proximal tubule, inhibition of SGLT2 restores solute release to the macula densa, restoring normal tubuloglomerular feedback, reversing afferent vasodilation, and normalizing glomerular hemodynamics [24].

Kidokoro et al. [25], studying renal hemodynamic alterations promoted by the use of empagliflozin in diabetic rats, demonstrated adenosine-dependent afferent arteriolar vasoconstriction with reduction of hyperfiltration within a few hours after drug administration.

On the other hand, the osmotic diuresis and the vasoconstriction of the afferent arteriole promoted by SGLT2i generate a concern regarding the possibility of dehydration and consequent AKI [9]. Although the exact mechanisms of acute kidney injury secondary to the use of iSGLT2 are not fully known, it is postulated that some factors related to the mechanism of action of this class may contribute to the acute worsening of renal function, such as, osmotic diuresis and glycosuria which can cause hyperosmolarity and dehydration. Increased levels of glucose in the urine can also be reabsorbed by the glucose transporter GLUT9b, located in the apical membrane of proximal tubular cells, in exchange for uric acid. Consequently, an increase in urinary uric acid level has also been proposed as a risk factor for AKI by crystal-dependent or crystal-independent mechanisms [21].

Food and Drug Administration (FDA) recommendation is for withdrawal of this class of drugs from 3 to days before a surgical procedure and it is based on these mechanisms [26]. Up to this date, there is no consensus on the maintenance or discontinuation of SGTL2i in patients who will undergo PCI.

With published data on the renal safety of empagliflozin pre-PCI in a pilot study, clinical outcomes such as CI-AKI can now be evaluated in a larger randomized clinical trial based on an adequate sample size calculation.

### Study limitations

This study has some limitations. 

SAFE-PCI is a pilot study, open label, with a relatively small sample size and a single-center trial which was not powered to assess clinical outcomes. CI-AKI was evaluated by a surrogate endpoint (serum NGAL) and not using a clinical definition for AKI. Other laboratory biomarkers such as L-FABP, NAG, albuminuria and urinary output were not measured due to early hospital discharge after elective and uncomplicated PCIs.

## Conclusion

The present study showed that the use of empagliflozin is safe regarding to kidney function during elective PCI in patients with T2D when compared to ones without SGLT2i. We did not observe significant changes after 6 h of PCI regarding serum NGAL levels, a biomarker of CI-AKI in this setting.

## Data Availability

The data of this trial is stored in REDCap–HCFMUSP program v11.0.3 (Vanderbilt University, Nashville, TN, USA).

## Abbreviations

ADA: American diabetes association
AKI: Acute kidney injury
BARC: Bleeding Academy Research Consortium
CABG: Coronary artery bypass graft
CAD: Coronary artery disease
CI-AKI: Contrast-induced acute kidney injury
eGFR: Estimated glomerular filtration rate
IQR: Interquartile ranges
KDIGO: Kidney Disease Improving Global Outcomes LVEF: left ventricular ejection fraction
MI: Myocardial infarction
NF-Kβ: Nuclear factor kappa-light-chain-enhancer of activated B cells, NGAL: Neutrophil Gelatinase-associated Lipocalin
PCI: Percutaneous coronary intervention
PMI: Periprocedural myocardial infarction
SCAI: Society for Cardiovascular Angiography and Interventions
SD: Standard deviation
SGLT2i: Sodium-glucose cotransporter 2 inhibitors
T2D: Type 2 diabetes
